# Isoserine Improves Spatial Memory and Remodels Synaptic and Inflammatory Gene-Expression Programs in APP/PS1 Mice

**DOI:** 10.3390/cells15161487

**Published:** 2026-08-18

**Authors:** Alessandra Saitta, Rossella Basilotta, Marika Lanza, Michele Scuruchi, Giovanna Casili, Pietro Giovani, Agata Copani, Emanuela Esposito, Salvatore Oddo, Antonella Caccamo

**Affiliations:** 1 Department of Chemical, Biological, Pharmaceutical and Environmental Sciences, University of Messina, Viale Ferdinando Stagno D’Alcontres 31, 98166 Messina, Italy; alessandra.saitta@studenti.unime.it (A.S.); rossella.basilotta@unime.it (R.B.); mlanza@unime.it (M.L.); gcasili@unime.it (G.C.); pietro.giovani@studenti.unime.it (P.G.); antonella.caccamo@unime.it (A.C.); 2 Department of Chemistry, Biology and Biotechnology, University of Perugia, Via Elce di Sotto 8, 06123 Perugia, Italy; 3 Department of Clinical and Experimental Medicine, University of Messina, Via Consolare Valeria 1, 98125 Messina, Italy; michele.scuruchi@unime.it; 4 Department of Drug and Health Sciences, University of Catania, 95125 Catania, Italy; caraless@unict.it

**Keywords:** Alzheimer’s disease, synaptic dysfunction, cognitive decline

## Abstract

Synaptic dysfunction is a major contributor to cognitive decline in Alzheimer’s disease (AD) and represents an attractive therapeutic target. Here, we investigated whether chronic isoserine treatment improves cognition and alters the expression of synaptic-related genes in APP/PS1 mice. Isoserine was well tolerated and did not adversely affect body weight. In the Morris water maze, isoserine improved probe-trial performance in APP/PS1 mice, significantly reducing latency to the first platform-location crossing, while time spent in the target quadrant showed a directionally consistent but non-significant increase. To identify molecular correlates, we profiled 84 synaptic-related genes using a targeted RT^2^ Profiler PCR Array. Gene-level factorial analyses identified several nominal treatment-associated effects, but no individual isoserine effect in APP/PS1 mice remained significant after false-discovery-rate correction. In contrast, module-level analyses identified False-discovery rate (FDR)-significant changes in NF-κB/inflammatory, synaptic-maintenance, and glutamatergic-signaling gene-expression modules, with significant genotype × treatment interactions for the NF-κB/inflammatory and synaptic-maintenance modules. Exploratory heatmap and principal component analyses further illustrated disease-context-dependent expression patterns. Western blot analyses showed that isoserine reduced nuclear factor kappa B (NF-κB p65) and NMDA receptor subunit GluN2B (GluN2B) and increased postsynaptic density protein 95 (PSD-95) levels in APP/PS1 mice. These findings suggest that isoserine improves spatial memory retention and coordinately remodels synaptic and inflammatory molecular programs in APP/PS1 mice.

## 1. Introduction

Alzheimer’s disease (AD) is characterized clinically by progressive cognitive decline and pathologically by amyloid-β (Aβ) deposition, tau pathology, neuroinflammation, and synaptic degeneration [1]. Although amyloid plaques and neurofibrillary tangles define the neuropathological diagnosis of AD, synaptic dysfunction and synapse loss correlate more closely with cognitive impairment than classical lesion burden alone [2,3,4,5]. These observations have focused increasing attention on mechanisms that preserve synaptic function and promote synaptic resilience, particularly during disease stages in which neuronal circuits may remain responsive to therapeutic modulation.

Synaptic dysfunction in AD arises from multiple converging mechanisms. Soluble Aβ assemblies impair long-term potentiation, alter dendritic spine structure, and disrupt hippocampal-dependent memory, independently of overt neuronal loss [6,7]. Aβ-associated synaptic toxicity also affects glutamatergic neurotransmission, calcium homeostasis, excitatory–inhibitory balance, and activity-dependent transcriptional programs required for memory encoding and consolidation [8,9]. Consistent with this framework, AD mouse models expressing familial AD-linked amyloid precursor protein (APP) and presenilin mutations develop amyloid pathology and cognitive deficits accompanied by abnormalities in synaptic signaling and plasticity [10,11,12]. These models therefore provide useful experimental systems to test whether candidate interventions can improve cognition and remodel synaptic molecular pathways in the context of amyloid-associated dysfunction.

Activity-dependent transcription is essential for experience-dependent synaptic plasticity and long-term memory formation. Activity-dependent regulators such as cAMP response element-binding protein and immediate early genes such as activity-regulated cytoskeleton-associated protein (Arc), early growth response 1 (Egr1), early growth response 2 (Egr2), jun proto-oncogene (Jun), and serum response factor (Srf) help link neuronal activity to persistent transcriptional and structural changes at synapses [13,14,15]. Disruption of these pathways may compromise the ability of neuronal circuits to adapt to experience and thereby contribute to cognitive impairment [16]. In parallel, synaptic maintenance proteins and glutamatergic signaling components, including PSD-95/DLG4, neuronal pentraxin-2 (NPTX2), and NMDA receptor subunits, organize postsynaptic signaling complexes and support excitatory synaptic function [17,18]. Alterations in these pathways are particularly relevant to AD because synaptic failure is a major determinant of memory decline.

Neuroinflammatory signaling also intersects with synaptic dysfunction in AD. Nuclear factor kappa B (NF-κB) signaling regulates inflammatory responses, cell survival pathways, and synaptic plasticity, and its effects vary according to cell type, activation context, and disease stage [19]. In the AD brain, chronic inflammatory activation can amplify neuronal vulnerability and synaptic dysfunction, whereas tightly regulated NF-κB signaling may also participate in adaptive neuronal responses. Thus, therapeutic strategies that modulate both synaptic and inflammatory transcriptional programs may be particularly relevant for restoring circuit function in disease.

Isoserine is a serine-related amino acid derivative whose biological effects have not previously been examined in an AD model. Previous work identified L-isoserine as a GAT3-preferring substrate and showed that delayed local administration increased GAT3 expression and improved functional recovery after focal ischemic stroke in mice [20]. However, this acute focal-injury setting differs from chronic amyloid-associated neurodegeneration and does not establish whether L-isoserine can improve cognition in AD. To our knowledge, no previous study has determined whether chronic L-isoserine treatment affects cognitive function in an AD model or whether any behavioral effects are accompanied by changes in synaptic and inflammatory gene-expression programs. It also remains unknown whether healthy and amyloid-bearing brains exhibit different molecular responses to L-isoserine.

**Figure 1:**
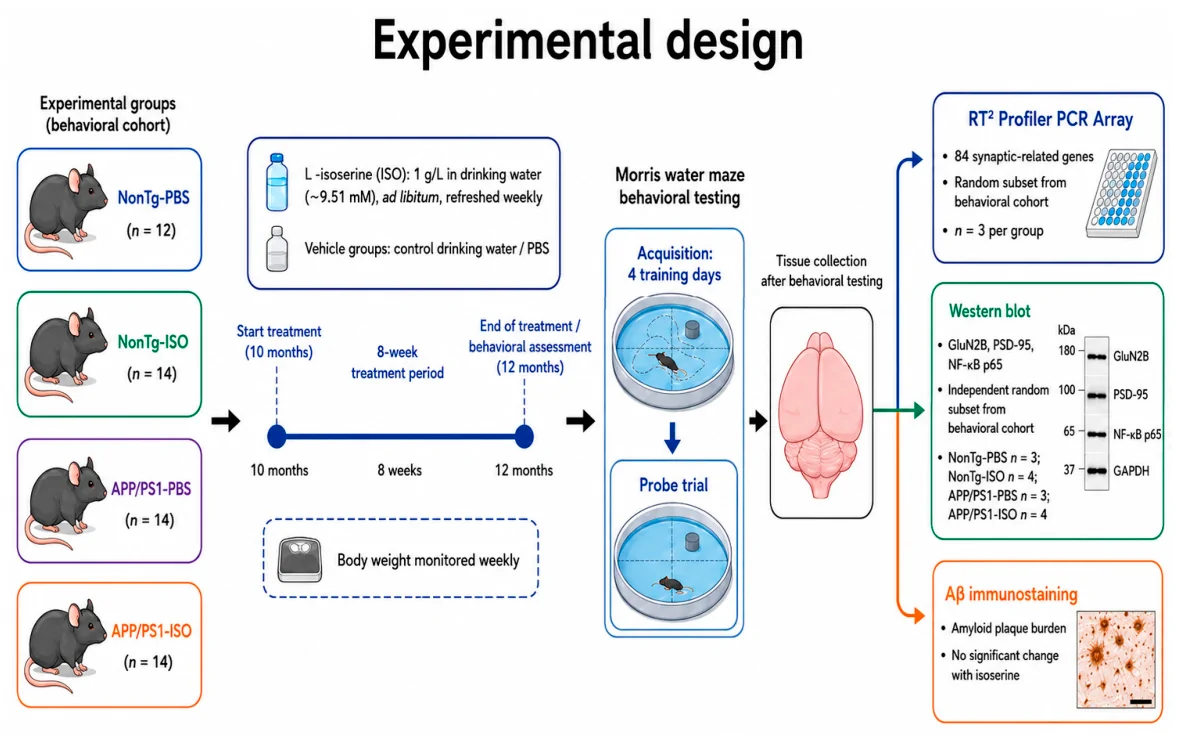
**Schematic representation of the experimental design.** NonTg and APP/PS1 mice received vehicle or L-isoserine in drinking water for 8 weeks, from 10 to 12 months of age. Body weight was monitored weekly, followed by Morris water maze testing and tissue collection. Randomly selected samples were analyzed by RT^2^ Profiler PCR Array, Western blot, and Aβ immunostaining.

To address these knowledge gaps, we investigated whether chronic L-isoserine treatment improves spatial memory and alters synaptic and inflammatory gene-expression programs in APP/PS1 mice and whether these molecular responses differ from those observed in NonTg animals (Figure 1). Hereafter, we use the terms isoserine and L-isoserine interchangeably. We assessed spatial learning and memory and measured the expression of 84 synaptic-related genes using an RT^2^ Profiler PCR Array. We integrated factorial gene-level analyses, effect-size visualizations, exploratory sample-level heatmap and principal component analyses, and functional module-level analyses. We further examined representative proteins related to glutamatergic signaling, postsynaptic organization, and inflammatory regulation. Our findings show that isoserine improves spatial-memory retention and induces coordinated disease-context-dependent remodeling of synaptic and inflammatory molecular programs in APP/PS1 mice.

## 2. Results

### 2.1. Isoserine Treatment Is Well-Tolerated in NonTg and APP/PS1 Mice

**Figure 2:**
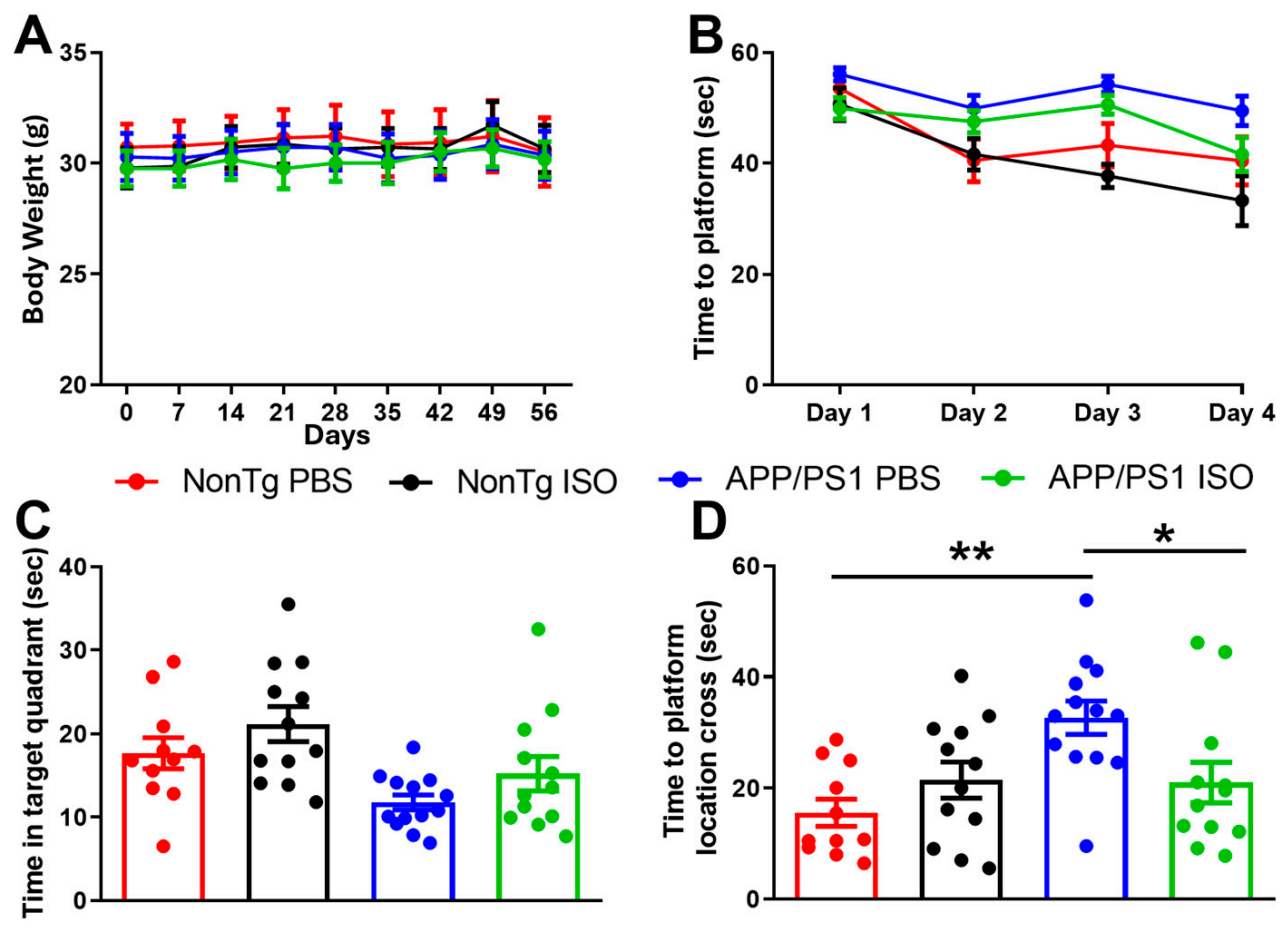
**Effects of chronic isoserine treatment on body weight, spatial learning and memory in NonTg and APP/PS1 mice.** (**A**) Body weight measured throughout the treatment period in NonTg-PBS (n = 12), NonTg-ISO (n = 14), APP/PS1-PBS (n = 14), and APP/PS1-ISO (n = 14) mice. (**B**) Escape latency during the four-day acquisition phase of the Morris water maze. Two-way repeated-measures ANOVA identified significant effects of training day [F(3, 192) = 11.13, *p* < 0.0001] and experimental group [F(3, 192) = 12.83, *p* < 0.0001], but no significant day × group interaction [F(9, 192) = 1.319, *p* = 0.2294]. (**C**) Time spent in the target quadrant during the probe trial differed among groups (one-way ANOVA, *p* = 0.0036). Tukey’s multiple-comparisons test showed that APP/PS1-PBS mice spent less time in the target quadrant than the NonTg control groups (*p* < 0.05); APP/PS1-ISO mice did not differ significantly from the NonTg groups. (**D**) Latency to the first crossing of the former platform location differed among groups (one-way ANOVA, *p* = 0.0030). Tukey’s multiple-comparisons test showed a difference between APP/PS1-PBS and NonTg-PBS mice (*p* < 0.01, indicated as ** in the figure) and a lower latency in APP/PS1-ISO than APP/PS1-PBS mice (*p* < 0.05, indicated as * in the figure). Data are presented as mean ± SEM.

To determine whether chronic isoserine (ISO) administration affected general health, we monitored body weight throughout the treatment period in mice treated with ISO or phosphate-buffered saline (PBS). Specifically, we randomly divided the mice in four different groups: NonTg-PBS (n = 12), NonTg-ISO (n = 14), APP/PS1-PBS (n = 14), and APP/PS1-ISO (n = 14) mice. Body weight remained stable during the experimental period, and neither NonTg nor APP/PS1 mice showed evidence of adverse treatment-related effects, indicating that chronic isoserine administration was well tolerated and did not cause overt systemic deterioration or treatment-associated weight loss under these experimental conditions (Figure 2A). These data indicate that the isoserine regimen was well-tolerated and support the interpretation that the behavioral and molecular effects observed in subsequent analyses did not result from treatment-induced deterioration in general health.

### 2.2. Isoserine Improves Spatial Memory Retention in APP/PS1 Mice

We next assessed hippocampal-dependent spatial learning and memory using the Morris water maze. During the acquisition phase of the Morris water maze, all groups improved across training days, as indicated by a significant effect of day (two-way repeated-measures ANOVA: F(3, 192) = 11.13, *p* < 0.0001; Figure 2B). We also detected a significant effect of experimental group (F(3, 192) = 12.83, *p* < 0.0001), indicating overall performance differences among groups. However, the day × group interaction did not reach statistical significance (F(9, 192) = 1.319, *p* = 0.2294), indicating that isoserine did not significantly modify the acquisition trajectory across training days.

We then evaluated spatial memory retention during the probe trial. Isoserine improved probe trial performance in APP/PS1 mice. In the target-quadrant analysis, one-way ANOVA revealed a significant group effect (Figure 2C; *p* = 0.0036). Tukey’s multiple comparisons test showed that APP/PS1-PBS mice spent significantly less time in the target quadrant than NonTg control groups (*p* < 0.05), whereas APP/PS1-ISO mice did not differ significantly from NonTg mice. We obtained stronger results when we analyzed the latency to the first platform-location crossing. One-way ANOVA revealed a significant group effect (Figure 2D; *p* = 0.0030). Tukey’s multiple comparisons test showed that APP/PS1-PBS mice differed significantly from NonTg-PBS mice (*p* < 0.01), whereas APP/PS1-ISO mice performed significantly better than APP/PS1-PBS mice (*p* < 0.05). Together, these findings indicate that isoserine improves spatial memory retention in APP/PS1 mice.

To determine whether chronic isoserine treatment altered Aβ load, we immunostained sections from each group (n = 3/group) with an Aβ42-specific antibody. Quantitative Aβ immunostaining showed no significant difference in plaque burden between APP/PS1-PBS and APP/PS1-ISO mice, indicating that the treatment-associated behavioral and molecular effects were not accompanied by a detectable change in amyloid plaque deposition (Supplementary Figure S1).

### 2.3. Exploratory Gene-Level Analysis of Genotype-Dependent Expression Patterns Following Isoserine Treatment

To determine whether isoserine modulates synaptic gene expression in a genotype-dependent manner, we analyzed 84 synaptic-related genes using the RT^2^ Profiler PCR Array. We normalized raw Ct values to the mean Ct of the housekeeping genes Actb, B2m, Gapdh, Gusb, and Hsp90ab1 and expressed normalized transcript abundance as −ΔCt. The functional categories and representative biological roles of the genes discussed throughout the manuscript are summarized in Table 1.

For each gene, we fitted a two-factor linear model that included genotype, treatment, and genotype × treatment. We then estimated the isoserine treatment effects separately in NonTg and APP/PS1 mice and adjusted the gene-level *p*-values using the Benjamini–Hochberg procedure within each contrast family. Nineteen genes reached nominal *p* < 0.05 for the isoserine effect in APP/PS1 mice; however, none remained significant after false-discovery-rate correction. Eleven genes showed nominal genotype × treatment interactions, and only Rgs2 remained significant after correction (q = 0.027). Supplementary Table S1 reports the complete effect estimates, 95% confidence intervals, raw *p*-values, and adjusted q-values for all genes and contrasts.

**Figure 3:**
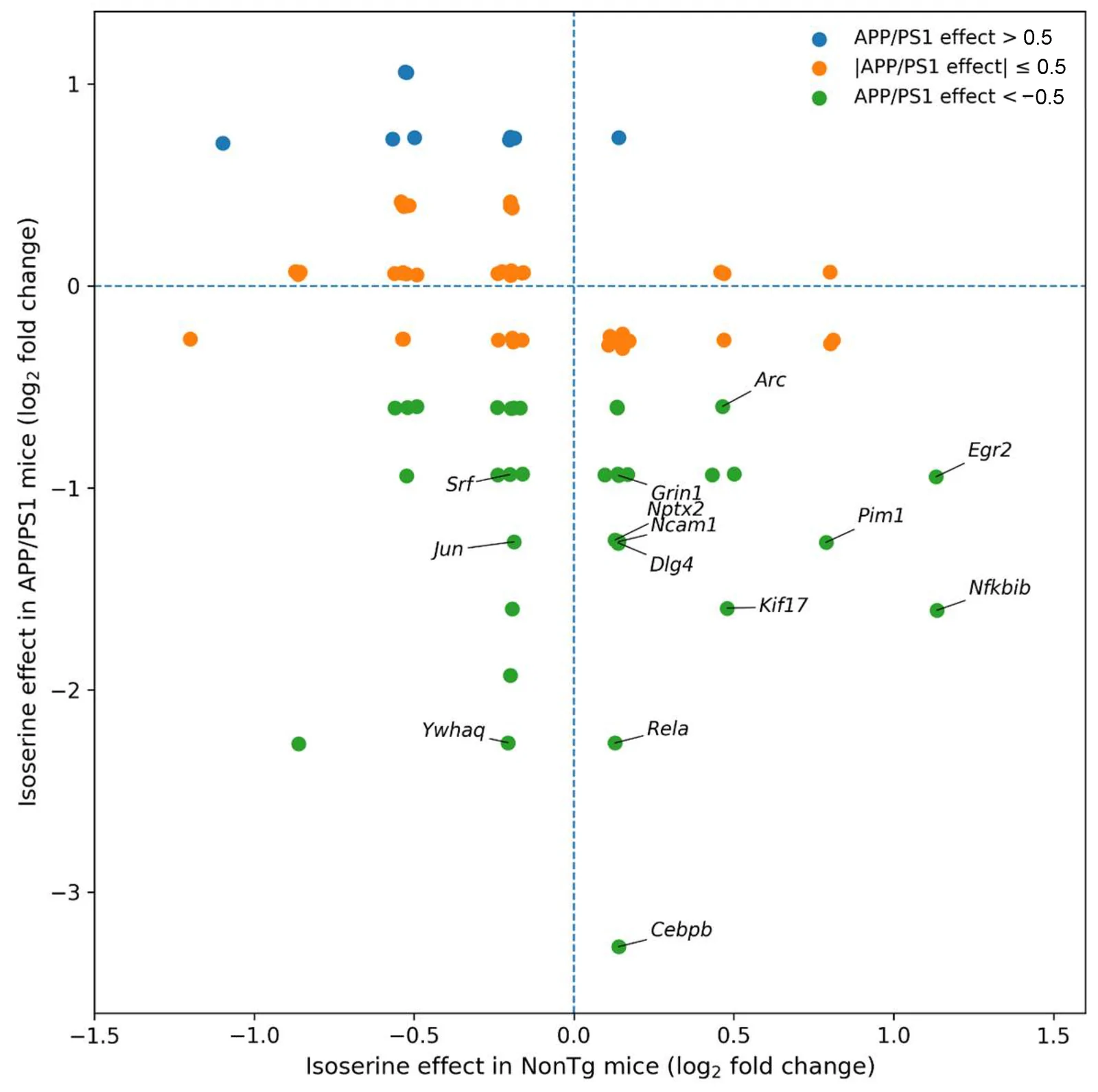
**Gene-level effect-size visualization of the transcriptional response to isoserine in NonTg and APP/PS1 mice**. Each point represents one of the 84 synaptic-related genes included in the RT^2^ Profiler PCR Array. The x-axis shows the isoserine treatment effect in NonTg mice, calculated as the log2 fold change between NonTg-ISO and NonTg-PBS groups. The y-axis shows the corresponding treatment effect in APP/PS1 mice, calculated as the log2 fold change between APP/PS1-ISO and APP/PS1-PBS groups. Positive values indicate higher expression after isoserine treatment, whereas negative values indicate lower expression. Dashed horizontal and vertical lines at zero indicate no treatment effect in APP/PS1 and NonTg mice, respectively. Genes near the origin showed limited treatment-associated changes in both genotypes, whereas genes farther from the origin showed larger expression effects. Differences between the x- and y-axis values indicate genotype-dependent responses to isoserine. Blue points identify genes with an APP/PS1 treatment effect greater than 0.5 log_2_ units, green points identify genes with an APP/PS1 treatment effect lower than −0.5 log_2_ units, and orange points identify genes with an absolute APP/PS1 treatment effect of 0.5 log_2_ units or less. Marker shape, fill, and size do not encode statistical significance. A small deterministic jitter was applied only to overlapping, unlabeled points to improve visualization and did not alter the underlying effect estimates. Selected genes were annotated because they showed comparatively large or genotype-dependent effects and represented biological processes related to inflammatory regulation, activity-dependent transcription, synaptic organization, glutamatergic signaling, and intracellular signaling. These genes include Cebpb, Nfkbib, Rela, Arc, Egr2, Jun, Srf, Dlg4, Ncam1, Nptx2, Grin1, Kif17, Pim1, and Ywhaq. No individual isoserine treatment effect in APP/PS1 mice remained significant after false-discovery-rate correction across the 84 genes; therefore, the figure should be interpreted as an effect-size visualization rather than as evidence of independently significant regulation of the annotated genes. To this end, gene annotation was performed post hoc for visual clarity and did not determine inclusion in any statistical analysis. Supplementary Table S1 reports the effect estimates, 95% confidence intervals, raw *p*-values, and Benjamini–Hochberg-adjusted q-values for all genes and contrasts.

We therefore used the treatment-effect scatter plot primarily to visualize effect sizes and genotype-dependent expression patterns rather than to identify independently significant genes (Figure 3). Several genes involved in inflammatory regulation, activity-dependent transcription, synaptic organization, and glutamatergic signaling showed comparatively large or genotype-dependent responses to isoserine. These genes included CCAAT/enhancer-binding protein beta (Cebpb), NF-kappa-B inhibitor beta (Nfkbib), RELA proto-oncogene, NF-kappa-B subunit (Rela), Arc, Egr2, Jun, Srf, discs large MAGUK scaffold protein 4 (Dlg4), neural cell adhesion molecule 1 (Ncam1), neuronal pentraxin 2 (Nptx2), glutamate ionotropic receptor NMDA type subunit 1 (Grin1), kinesin family member 17 (Kif17), Pim-1 proto-oncogene, serine/threonine kinase (Pim1), and tyrosine 3-monooxygenase/tryptophan 5-monooxygenase activation protein theta (Ywhaq). We highlighted these genes because of their magnitude of effect, genotype-dependent patterns, and biological relevance, but we interpret their individual responses as exploratory.

Together, these findings show that NonTg and APP/PS1 mice mount different transcriptional responses to isoserine. However, the absence of FDR-significant treatment effects at the individual-gene level prevents us from identifying specific genes as independently confirmed isoserine targets.

### 2.4. Directional Relationship Between Disease- and Isoserine-Associated Gene-Expression Effects

**Figure 4:**
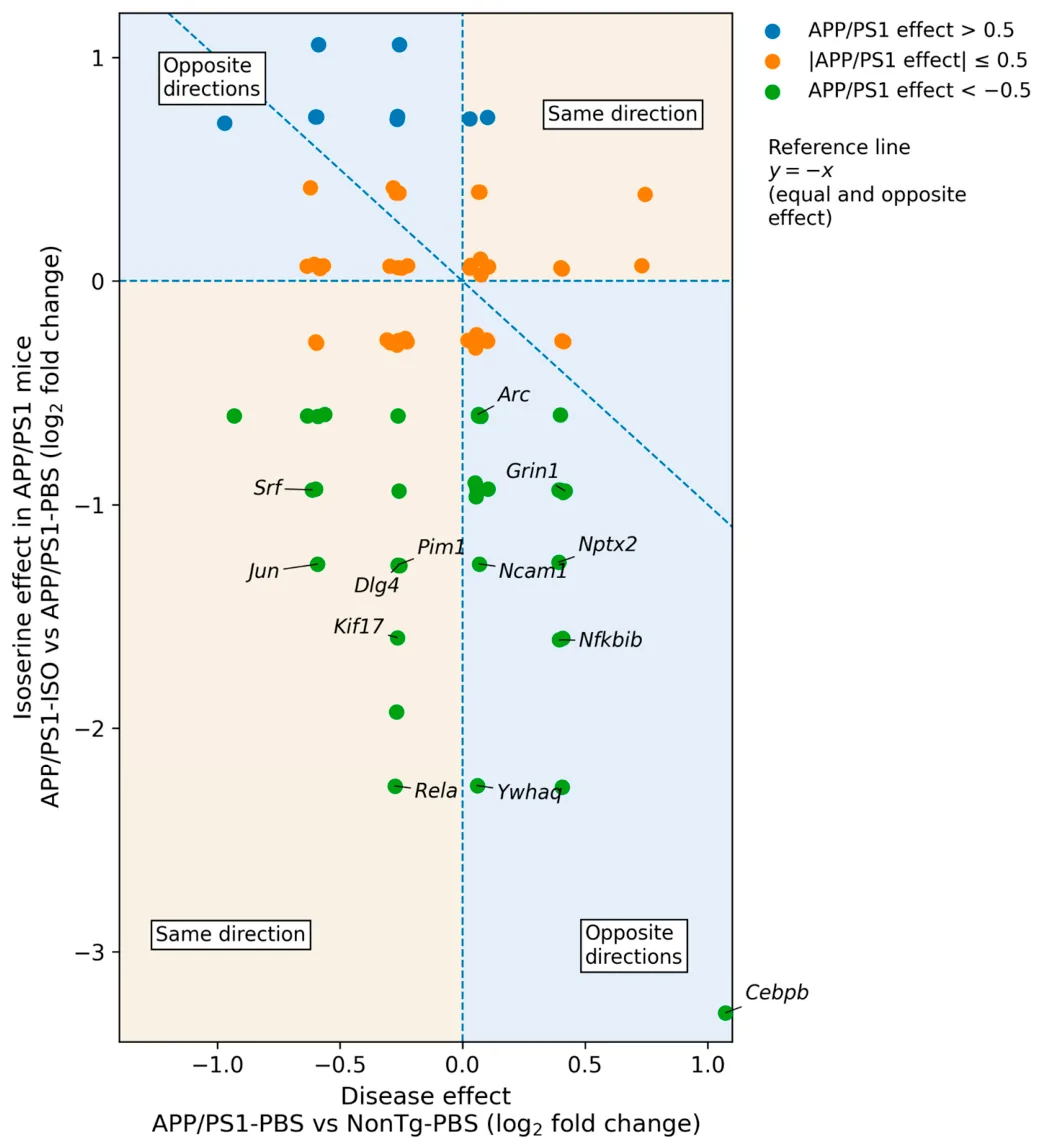
**Descriptive relationship between disease- and isoserine-associated gene-expression effects.** Each point represents one of the synaptic-related genes included in the RT^2^ Profiler PCR Array. The x-axis shows the disease effect, calculated as the log2 fold change between APP/PS1-PBS and NonTg-PBS mice. Positive x-axis values indicate higher expression in APP/PS1-PBS mice than in NonTg-PBS controls, whereas negative values indicate lower expression. The y-axis shows the isoserine treatment effect in APP/PS1 mice, calculated as the log2 fold change between APP/PS1-ISO and APP/PS1-PBS groups. Positive y-axis values indicate higher expression after isoserine treatment, whereas negative values indicate lower expression. Dashed horizontal and vertical lines at zero indicate no treatment effect and no disease effect, respectively. Genes in the upper-left and lower-right quadrants showed treatment-associated changes in the direction opposite to the corresponding disease-associated effects, whereas genes in the upper-right and lower-left quadrants showed disease and treatment effects in the same direction. The dashed diagonal line, y = −x, represents an equal and opposite effect and is included only as a visual reference. Blue points identify genes with an APP/PS1 treatment effect greater than 0.5 log_2_ units, green points identify genes with an APP/PS1 treatment effect lower than −0.5 log_2_ units, and orange points identify genes with an absolute APP/PS1 treatment effect of 0.5 log_2_ units or less. Marker shape, fill, and size do not encode statistical significance. A small deterministic jitter was applied only to overlapping, unlabeled points to improve visualization and did not alter the underlying effect estimates. Selected genes were annotated because they showed prominent directional patterns, comparatively large effects, or biological relevance to inflammatory regulation, synaptic maintenance, glutamatergic signaling, activity-dependent transcription, and intracellular signaling. Because the disease and treatment contrasts both include the APP/PS1-PBS group with opposite signs, the relationship shown in the plot was interpreted descriptively and was not used as an inferential test of transcriptional rescue or global normalization. Supplementary Table S1 reports the effect estimates, 95% confidence intervals, raw *p*-values, and Benjamini–Hochberg-adjusted q-values for all genes and contrasts.

We next examined whether isoserine shifted disease-associated gene-expression changes in the opposite direction. For each gene, we calculated the disease effect as the log_2_ fold change between APP/PS1-PBS and NonTg-PBS mice and the treatment effect as the log_2_ fold change between APP/PS1-ISO and APP/PS1-PBS mice. Genes located in the upper-left and lower-right quadrants of the scatter plot showed treatment-associated changes that opposed the corresponding disease-associated effects, whereas genes in the other two quadrants showed changes in the same direction (Figure 4).

Several genes associated with inflammatory regulation, synaptic maintenance, glutamatergic signaling, and activity-dependent transcription exhibited opposite disease and treatment effects. These genes included Cebpb, Nfkbib, Nptx2, Grin1, and Ncam1. Other genes, including Rela, Kif17, Pim1, Dlg4, Jun, and Srf, showed more complex directional patterns. These observations illustrate that isoserine did not produce a uniform shift across the targeted gene panel.

Because both contrasts included the APP/PS1-PBS group, with opposite signs, we treated the relationship between the disease and treatment effects as descriptive rather than inferential. We therefore did not use the gene-wise correlation as a statistical test of transcriptional rescue or global normalization. Instead, Figure 4 illustrates the direction and magnitude of the expression effects and complements the FDR-adjusted module-level analyses described below.

### 2.5. Selected Genes Illustrate Sample-Level Expression Patterns Following Isoserine Treatment

To illustrate sample-level expression patterns among genes highlighted by the effect-size and functional analyses, we generated a heatmap using normalized expression values for 14 genes. Gene selection was based on effect magnitude, genotype-dependent expression patterns, representation of the functional modules, and biological relevance to synaptic and inflammatory processes. Because the genes were selected after examination of the dataset, the heatmap was considered exploratory and was not used as an independent statistical test.

**Figure 5:**
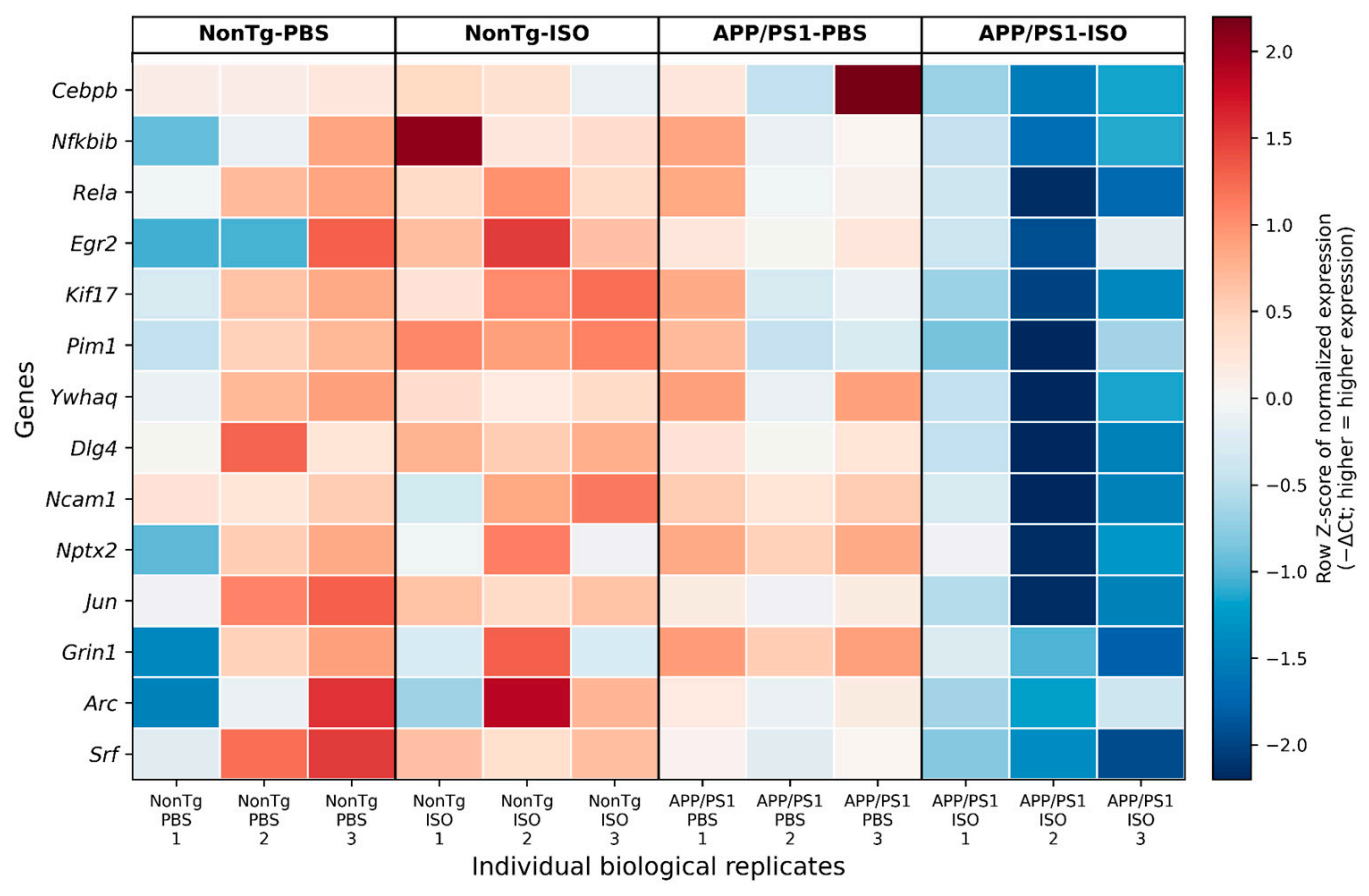
**Selected genes illustrate sample-level expression patterns across the four experimental groups.** The heatmap displays sample-level normalized expression values for 14 genes selected based on effect magnitude, genotype-dependent expression patterns, representation of the predefined functional modules, and biological relevance to synaptic and inflammatory processes. The selected genes include Cebpb, Nfkbib, Rela, Egr2, Kif17, Pim1, Ywhaq, Dlg4, Ncam1, Nptx2, Jun, Grin1, Arc, and Srf. Columns represent individual biological replicates from the NonTg-PBS, NonTg-ISO, APP/PS1-PBS, and APP/PS1-ISO groups (n = 3 per group), and vertical black lines separate the experimental groups. Raw Ct values were normalized within each sample to the mean Ct of the housekeeping genes Actb, B2m, Gapdh, Gusb, and Hsp90ab1. Relative transcript abundance was expressed as −ΔCt, with higher values indicating greater expression. Each gene was then standardized across the 12 biological samples and displayed as a row-wise Z-score. Red tones indicate expression above the across-sample mean for a given gene, whereas blue tones indicate expression below that mean. The color scale therefore shows relative variation within each gene and does not permit direct comparison of absolute expression levels between different genes. APP/PS1-ISO samples showed expression patterns that differed from APP/PS1-PBS samples across genes related to inflammatory regulation, synaptic organization, glutamatergic signaling, intracellular signaling, and activity-dependent transcription. Because the genes were selected after examination of the dataset, the heatmap provides an exploratory visualization of sample-level patterns and does not constitute an independent statistical test of differential expression or normalization. No prespecified statistical threshold was used to select the displayed genes. Gene-level effect estimates, 95% confidence intervals, raw *p*-values, and Benjamini–Hochberg-adjusted q-values are reported in Supplementary Table S1.

The heatmap showed that NonTg-ISO samples generally resembled NonTg-PBS samples, consistent with the relatively modest transcriptional response to isoserine in healthy animals (Figure 5). In contrast, APP/PS1-ISO samples differed clearly from APP/PS1-PBS samples across multiple genes. This treatment-associated pattern appeared particularly evident for transcripts involved in NF-κB/inflammatory signaling, including Cebpb, Nfkbib, and Rela, and for synaptic maintenance genes, including Dlg4, Ncam1, and Nptx2. Genes linked to glutamatergic signaling, intracellular signaling, and activity-dependent transcription also showed treatment-associated changes.

Because we performed this analysis at the level of individual biological replicates rather than group means, the heatmap provides sample-level support for the transcriptional remodeling observed in the scatter-plot analyses. These findings indicate that isoserine modulates a biologically coherent set of synaptic and inflammatory genes in APP/PS1 mice.

### 2.6. Exploratory Principal Component Analysis Illustrates Group-Associated Expression Variation

To determine whether isoserine influenced the global transcriptional structure of the dataset, we performed principal component analysis using sample-level normalized expression values from the RT^2^ Profiler PCR Array. We excluded housekeeping genes and technical control wells from the analysis. Of the 84 synaptic-related target genes, 83 remained after removal of genes with missing or zero-variance values incompatible with PCA.

**Figure 6:**
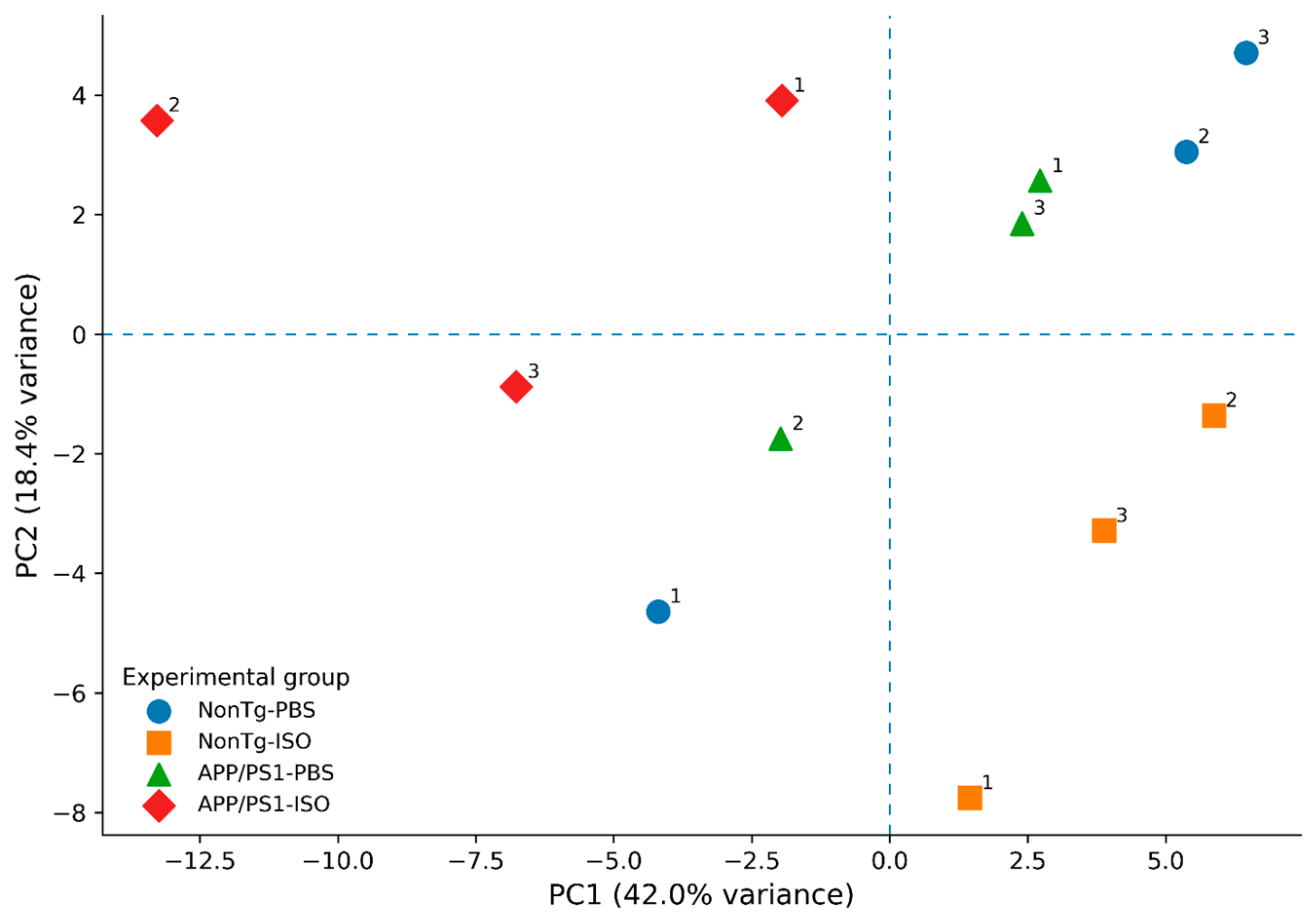
**Exploratory principal component analysis of synaptic gene-expression profiles.** Each point represents one individual mouse, and different symbols indicate the four experimental groups: NonTg-PBS, NonTg-ISO, APP/PS1-PBS, and APP/PS1-ISO. PC1 accounted for 42.0% of the total variance, whereas PC2 accounted for 18.4%; together, PC1 and PC2 explained 60.3% of the total variance. The PCA showed genotype- and treatment-associated variation among samples. APP/PS1-PBS mice occupied a region that differed from several NonTg samples in the first two principal components. APP/PS1-ISO samples occupied a different region from APP/PS1-PBS samples, consistent with treatment-associated variation in the targeted expression profile. APP/PS1-ISO samples did not uniformly overlap with NonTg samples; however, PCA was not used to infer treatment-induced normalization. PCA was used descriptively to visualize major sources of variation; it was not used to test the statistical significance of separation among groups.

PC1 explained 42.0% of the total variance, whereas PC2 explained 18.4%, together accounting for 60.3% of the variance in the dataset (Figure 6). The plotted samples showed genotype- and treatment-associated variation, with APP/PS1-ISO samples occupying a region that differed from APP/PS1-PBS samples. However, because the analysis included only three biological replicates per group and substantially more variables than samples, PCA was used exclusively as an exploratory visualization. No inferential test of group separation was performed, and the PCA should not be interpreted as evidence of statistically established global normalization or treatment-induced clustering.

### 2.7. Module-Level Analysis Identifies Coordinated Changes in Synaptic and Inflammatory Gene-Expression Modules

We next examined whether biologically related genes showed coordinated expression changes following isoserine treatment. We performed an exploratory module-level analysis using four literature-informed gene-expression modules that represented biological domains directly relevant to synaptic function and inflammatory regulation. These modules were not preregistered as confirmatory endpoints before examination of the treatment dataset. We defined their composition according to established biological functions and fixed the gene assignments before performing the formal module-level statistical analyses.

The immediate-early-gene module included Arc, Egr1, Egr2, Jun, and Srf, which participate in activity-dependent transcription and synaptic plasticity. The glutamatergic-signaling module included glutamate ionotropic receptor AMPA type subunit 4 (Gria4), Grin1, glutamate ionotropic receptor NMDA type subunit 2b (Grin2b), and glutamate metabotropic receptor 1 (Grm1), representing AMPA, NMDA, and metabotropic glutamate receptor signaling. The synaptic-maintenance module included Dlg4, Ncam1, and Nptx2, which contribute to postsynaptic organization, cell adhesion, and excitatory synaptic maintenance. The NF-κB/inflammatory module included Cebpb, Rela, and Nfkbib, which participate in NF-κB-associated transcriptional and inflammatory regulation.

We did not assign genes with broad, pleiotropic, or ambiguous functions to these modules and did not require all genes represented on the array to enter the module analysis. Each included gene contributed to only one module. For each biological sample, we calculated the module score as the arithmetic mean of the −ΔCt values of the constituent genes. We weighted all genes equally and did not use weights based on expression abundance, variance, fold change, or statistical significance.

We applied the same factorial model used for the individual-gene analyses to each module score and estimated the disease effect, the isoserine treatment effect in NonTg mice, the isoserine treatment effect in APP/PS1 mice, and the genotype × treatment interaction. We then applied the Benjamini–Hochberg procedure across the four modules separately within each contrast family.

**Figure 7:**
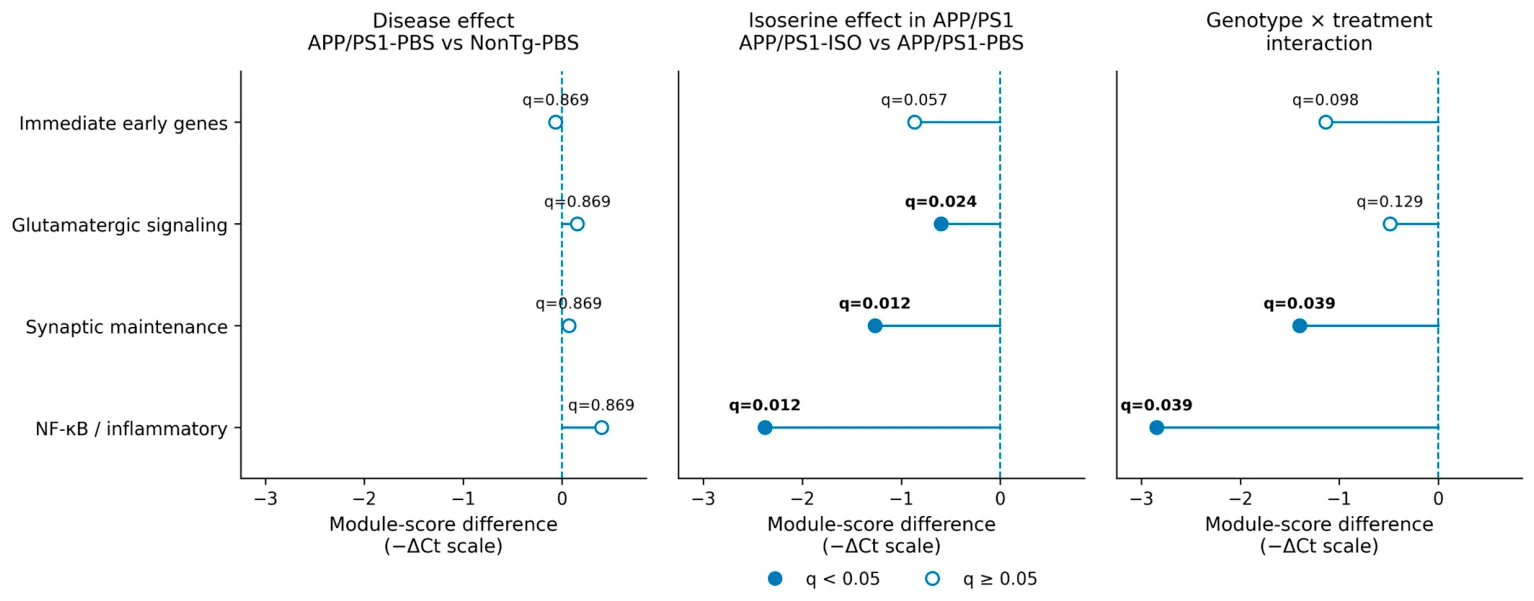
**Module-level analysis of treatment-associated changes in synaptic and inflammatory gene-expression modules.** Lollipop plots summarize the disease effects, the isoserine treatment effects in APP/PS1 mice, and genotype × treatment interactions for four literature-informed exploratory gene expression modules derived from the RT^2^ Profiler PCR Array. The immediate early genes module included Arc, Egr1, Egr2, Jun, and Srf; the glutamatergic signaling module included Gria4, Grin1, Grin2b, and Grm1; the synaptic maintenance module included Dlg4, Ncam1, and Nptx2; and the NF-κB/inflammatory module included Cebpb, Rela, and Nfkbib. Module scores represent the equal-weight mean of the sample-level −ΔCt values for the genes assigned to each module. The left panel shows the disease effect, calculated as APP/PS1-PBS minus NonTg-PBS mice. The middle panel shows the isoserine treatment effect in APP/PS1 mice, calculated as APP/PS1-ISO minus APP/PS1-PBS. The right panel shows the genotype × treatment interaction effect, calculated as the difference between isoserine treatment effect in APP/PS1 and the corresponding effect in NonTg mice. Points indicate estimated module-level effects, and horizontal lines extend from zero to the corresponding effect size. Benjamini–Hochberg correction was applied across the four modules separately within each contrast family. Filled circles indicate effects that remained significant after FDR correction (q < 0.05), whereas open circles indicate effects with q ≥ 0.05. In APP/PS1 mice, isoserine significantly reduced the NF-κB/inflammatory module score (effect = −2.38, 95% CI, −3.80 to −0.96; *p* = 0.0048, q = 0.0119), the synaptic-maintenance module score (effect = −1.26, 95% CI, −2.05 to −0.48; *p* = 0.0059, q = 0.0119), and the glutamatergic-signaling module score (effect = −0.60, 95% CI, −1.07 to −0.13; *p* = 0.0182, q = 0.0242). The immediate-early-gene module did not reach the FDR-adjusted threshold (effect = −0.87, 95% CI, −1.77 to 0.03; *p* = 0.0566, q = 0.0566). Significant genotype × treatment interactions occurred for the NF-κB/inflammatory module (interaction effect = −2.85, 95% CI, −4.85 to −0.84; *p* = 0.0114, q = 0.0393) and the synaptic-maintenance module (interaction effect = −1.40, 95% CI, −2.51 to −0.29; *p* = 0.0197, q = 0.0393). Supplementary Table S2 reports the complete module-level effect estimates, 95% confidence intervals, raw *p*-values, and FDR-adjusted q-values.

In APP/PS1 mice, isoserine significantly reduced the NF-κB/inflammatory module score (effect = −2.38, 95% CI, −3.80 to −0.96; *p* = 0.0048, q = 0.0119), the synaptic-maintenance module score (effect = −1.26, 95% CI, −2.05 to −0.48; *p* = 0.0059, q = 0.0119), and the glutamatergic-signaling module score (effect = −0.60, 95% CI, −1.07 to −0.13; *p* = 0.0182, q = 0.0242; Figure 7). Isoserine also reduced the immediate-early-gene module score, but this effect did not reach the FDR-adjusted significance threshold (effect = −0.87, 95% CI, −1.77 to 0.03; *p* = 0.0566, q = 0.0566).

The genotype x treatment analysis showed that APP/PS1 and NonTg mice responded differently to isoserine in the NF-κB/inflammatory module (interaction effect = −2.85, 95% CI, −4.85 to −0.84; *p* = 0.0114, q = 0.0393) and the synaptic-maintenance module (interaction effect = −1.40, 95% CI, −2.51 to −0.29; *p* = 0.0197, q = 0.0393). The genotype × treatment interactions did not remain significant after FDR correction for the immediate-early-gene module (q = 0.0975) or the glutamatergic-signaling module (q = 0.1288).

These results show that isoserine coordinately modulated functional gene-expression programs related to inflammatory regulation, synaptic maintenance, and glutamatergic signaling in APP/PS1 mice. Importantly, these module-level effects remained significant after correction across the four literature-informed exploratory modules, despite the absence of FDR-significant APP/PS1 treatment effects at the individual-gene level. Supplementary Table S2 reports the complete module-level effect estimates, 95% confidence intervals, raw *p*-values, and adjusted q-values.

### 2.8. Protein-Level Support for Synaptic and Inflammatory Molecular Remodeling

**Figure 8:**
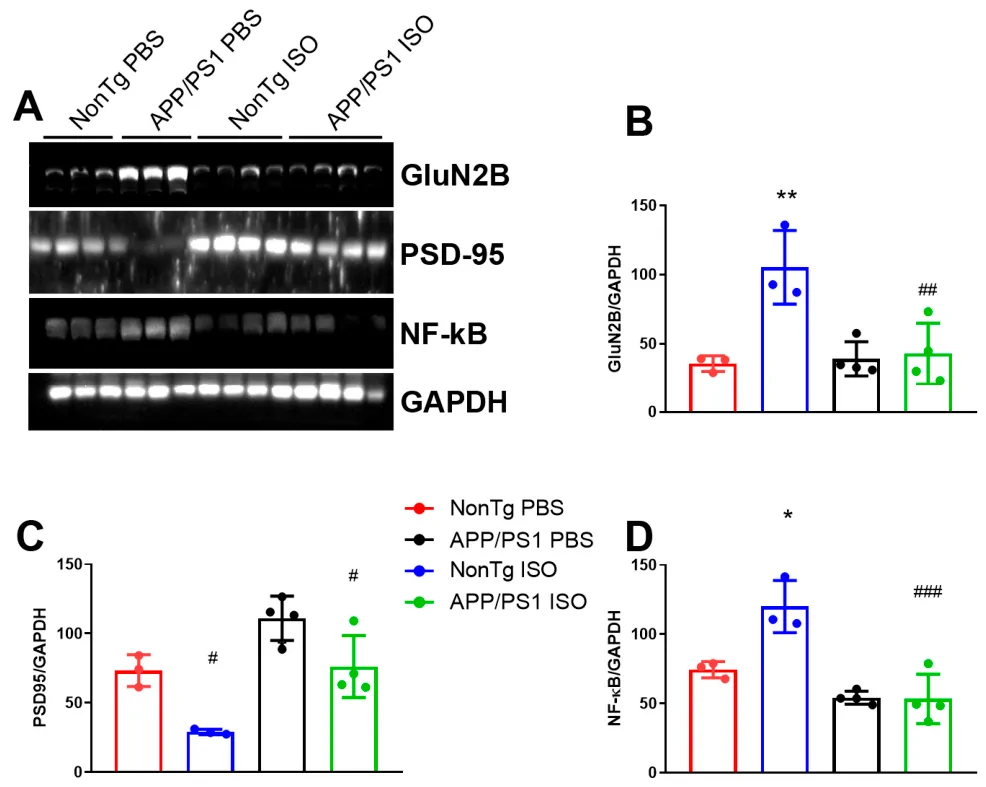
**Effects of isoserine on GluN2B, PSD-95, and NF-κB p65 protein abundance in APP/PS1 mice.** (**A**) Representative immunoblots show GluN2B, PSD-95, NF-κB p65, and GAPDH in NonTg-PBS, NonTg-ISO, APP/PS1-PBS, and APP/PS1-ISO mice. Protein levels were normalized to GAPDH. (**B**) Quantification of GluN2B showed a significant group effect (one-way ANOVA: F(3,10) = 10.1, *p* = 0.002, R^2^ = 0.751). Bonferroni’s multiple comparisons test showed that APP/PS1-PBS mice had significantly higher GluN2B levels than NonTg-PBS mice (*p* = 0.006). Isoserine did not alter GluN2B levels in NonTg mice (NonTg-ISO versus NonTg-PBS, *p* > 0.99), but significantly reduced GluN2B levels in APP/PS1 mice (APP/PS1-ISO versus APP/PS1-PBS, *p* = 0.008). APP/PS1-ISO mice no longer differed from NonTg-PBS or NonTg-ISO mice (both *p* > 0.99). (**C**) Quantification of PSD-95 also revealed a significant group effect (one-way ANOVA: F(3,10) = 15.1, *p* < 0.001, R^2^ = 0.819). APP/PS1-PBS mice showed significantly lower PSD-95 levels than NonTg-PBS mice (*p* = 0.04). Isoserine did not significantly alter PSD-95 levels in NonTg mice (NonTg-ISO versus NonTg-PBS, *p* = 0.07), but significantly increased PSD-95 levels in APP/PS1 mice (APP/PS1-ISO versus APP/PS1-PBS, *p* = 0.02). APP/PS1-ISO mice no longer differed from NonTg-PBS mice (*p* > 0.99) or NonTg-ISO mice (*p* = 0.07). (**D**) Quantification of NF-κB p65 showed a significant group effect (one-way ANOVA: F(3,10) = 17.8, *p* < 0.001, R^2^ = 0.842). APP/PS1-PBS mice showed significantly higher NF-κB p65 levels than NonTg-PBS mice (*p* = 0.01). Isoserine did not significantly affect NF-κB p65 levels in NonTg mice (NonTg-ISO versus NonTg-PBS, *p* = 0.46), but strongly reduced NF-κB p65 levels in APP/PS1 mice (APP/PS1-ISO versus APP/PS1-PBS, *p* < 0.001). APP/PS1-ISO mice no longer differed from NonTg-PBS mice (*p* = 0.40) or NonTg-ISO mice (*p* > 0.99). Brown–Forsythe tests did not detect significant differences in variance for GluN2B (*p* = 0.58), PSD-95 (*p* = 0.62), or NF-κB p65 (*p* = 0.65). Graphs show mean ± SEM. * *p* < 0.05, ** *p* < 0.01 vs. NonTg-PBS; # *p* < 0.05, ## *p* < 0.01, ### *p* < 0.001 vs. APP/PS1-PBS. Supplementary Table S3 reports the complete Western blot sample sizes, descriptive statistics, and effect estimates.

To determine whether protein-level changes accompanied the expression patterns identified by the RT^2^ Profiler PCR Array, we measured GluN2B, PSD-95, and NF-κB p65 by Western blot. We analyzed NonTg-PBS (n = 3), NonTg-ISO (n = 4), APP/PS1-PBS (n = 3), and APP/PS1-ISO (n = 4) for each protein and normalized all measurements to GAPDH. Brown–Forsythe tests detected no significant differences in variance for GluN2B (p=0.583), PSD-95 (p=0.622), or NF-κB p65 (*p* = 0.649; Figure 8).

One-way ANOVA identified a significant group effect for GluN2B ($F_{3,10}\;=\;10.06$, p = 0.0023, $R^{2\;}=\;0.751$). APP/PS1-PBS mice showed higher GluN2B levels than NonTg-PBS mice, with a mean difference of 69.73 normalized units (Bonferroni-adjusted 95% CI, 20.28 to 119.18; adjusted p = 0.0057). Isoserine did not significantly alter GluN2B in NonTg mice (mean difference, 3.47; 95% CI, −42.79  to 49.73; adjusted p > 0.99). In contrast, isoserine reduced GluN2B in APP/PS1 mice by 62.47 units (95% CI, −108.73 to −16.21; adjusted p = 0.0077). After treatment, APP/PS1-ISO mice did not differ from NonTg-PBS mice (mean difference, 7.26; 95% CI, −39.00 to 53.51; adjusted p > 0.99) or NonTg-ISO mice (mean difference, 3.79; 95% CI, −39.04 to 46.61; adjusted p > 0.99).

One-way ANOVA also identified a significant group effect for PSD-95 ($F_{3,10}\;=\;15.13$, p<0.001, $R^{2}\;=\;0.819$). APP/PS1-PBS mice showed lower PSD-95 levels than NonTg-PBS mice, with a mean difference of −44.25 units (95% CI, −86.90 to −1.60; adjusted p = 0.0406). Isoserine increased PSD-95 in NonTg mice by 37.72 units, although this comparison did not reach the adjusted significance threshold (95% CI, −2.18 to 77.61; adjusted p = 0.0677). In APP/PS1 mice, isoserine increased PSD-95 by 47.13 units (95% CI, 7.23 to 87.02; adjusted p  =  0.0186). Following treatment, APP/PS1-ISO mice did not differ from NonTg-PBS mice (mean difference, 2.88; 95% CI, −37.02 to 42.77; adjusted p > 0.99). The difference between APP/PS1-ISO and NonTg-ISO mice also did not reach the adjusted threshold (mean difference, −34.84; 95% CI, −71.77 to 2.09; adjusted p = 0.0685).

Finally, one-way ANOVA identified a significant group effect for NF-κB p65 ($F_{3,10}\;=\;17.79$, p<0.001, $R^{2}\;=\;0.842$). APP/PS1-PBS mice showed higher NF-κB p65 levels than NonTg-PBS mice, with a mean difference of 45.59 units (95% CI, 9.75 to 81.43; adjusted p = 0.0115). Isoserine did not significantly alter NF-κB p65 in NonTg mice (mean difference, −20.16; 95% CI, −53.68 to 13.37; adjusted p = 0.4627). In APP/PS1 mice, isoserine reduced NF-κB p65 by 66.65 units (95% CI, −100.17 to −33.12; adjusted p < 0.001). Following treatment, APP/PS1-ISO mice did not differ significantly from NonTg-PBS mice (mean difference, −21.06; 95% CI, −54.58 to 12.47; adjusted p = 0.3996) or NonTg-ISO mice (mean difference, −0.90; 95% CI, −31.94 to 30.14; adjusted p > 0.99). Supplementary Table S3 reports the complete Western blot sample sizes, descriptive statistics, and effect estimates. Together, these analyses provide complementary protein-level evidence that isoserine affects glutamatergic signaling, postsynaptic organization, and inflammatory regulation in APP/PS1 mice. The reductions in *Grin2b*/GluN2B and *Rela*/NF-κB p65 were directionally concordant across transcript and protein measurements. In contrast, *Dlg4* transcript abundance and PSD-95 protein abundance changed in opposite directions, indicating that the PSD-95 result supports altered postsynaptic protein abundance rather than direct directional validation of the *Dlg4* transcript effect.

## 3. Discussion

In this study, we investigated the behavioral and molecular effects of chronic isoserine treatment in APP/PS1 mice. We found that isoserine was well-tolerated and improved spatial memory retention in the Morris water maze, as shown by reduced latency to the first platform-location crossing during the probe trial. At the molecular level, isoserine induced a genotype-dependent transcriptional response across synaptic-related genes. The descriptive directionality analysis showed that several disease-associated expression effects were accompanied by oppositely directed treatment effects, whereas FDR-adjusted module-level analyses identified coordinated changes in NF-κB/inflammatory, synaptic-maintenance, and glutamatergic-signaling gene-expression modules. Western blot analyses provided complementary protein-level evidence, showing reduced NF-κB p65 and GluN2B abundance and increased PSD-95 abundance in isoserine-treated APP/PS1 mice. Together, these findings suggest that isoserine improves spatial memory retention and remodels selected synaptic and inflammatory molecular programs in a disease-context-dependent manner.

The behavioral findings provide the functional framework for interpreting the molecular results. During acquisition, all groups improved across training days and showed overall group differences, but the day × group interaction did not reach significance. Therefore, we do not interpret these data as evidence that isoserine significantly changed the acquisition trajectory. Instead, the strongest behavioral evidence comes from the probe trial, where isoserine improved spatial memory retention in APP/PS1 mice. This distinction is important because the probe trial more directly assesses memory for the platform location after training, whereas acquisition curves reflect multiple factors, including learning rate, search strategy, motivation, swimming efficiency, and repeated task exposure [21,22]. Thus, the behavioral data support the conclusion that isoserine improves spatial memory retention rather than broadly enhancing all aspects of water maze performance.

The transcriptional analyses provide a molecular framework for this behavioral improvement. The treatment-effect scatter plot showed that isoserine did not produce a uniform response across genotypes. Instead, several genes displayed stronger or qualitatively distinct regulation in APP/PS1 mice compared to NonTg animals. This disease-context dependence is biologically meaningful. The APP/PS1 brain contains amyloid-associated synaptic stress, inflammatory activation, and altered neuronal network function, which may create a molecular environment that responds differently to treatment than the healthy brain [9,23,24,25]. In this context, the broader transcriptional response observed in APP/PS1 mice may reflect the ability of isoserine to engage pathways that are selectively dysregulated or sensitized under disease conditions.

The gene-level analysis identified several comparatively large and nominally significant treatment-associated expression effects. However, no individual isoserine effect in APP/PS1 mice remained significant after FDR correction across the 84 target genes. This result is important when interpreting the molecular findings. Individual genes highlighted in the effect-size plots and heatmap should be viewed as exploratory contributors to broader expression patterns rather than as independently confirmed treatment targets. The absence of FDR-significant individual-gene effects likely reflects a combination of modest molecular sample size, biological variability, and the number of genes tested.

The descriptive directionality plot showed that several disease-associated expression effects were accompanied by oppositely directed treatment effects. Nevertheless, because the disease and treatment contrasts share the APP/PS1-PBS group, this relationship was not interpreted as a formal statistical demonstration of transcriptional rescue. The principal gene-expression evidence instead derives from the FDR-adjusted module analyses, which test whether coordinated groups of biologically related genes change collectively.

The exploratory heatmap visualized sample-level expression patterns for 14 genes selected after examination of the dataset based on effect magnitude, genotype-dependent patterns, module representation, and biological relevance. These genes included Cebpb, Nfkbib, Rela, Dlg4, Ncam1, Nptx2, Grin1, Kif17, Pim1, Ywhaq, Arc, Egr2, Jun, and Srf. The plot showed visually different patterns between APP/PS1-ISO and APP/PS1-PBS samples across genes related to inflammatory regulation, synaptic maintenance, intracellular signaling, glutamatergic signaling, and activity-dependent transcription. Because gene selection was post hoc and the analysis included only three animals per group, the heatmap does not establish reproducibility, differential expression, or treatment-induced normalization. Although these genes do not represent a single pathway, they converge on biological processes highly relevant to AD, including synaptic organization, excitatory neurotransmission, neuronal activity-dependent transcription, and inflammatory regulation [13,14,20,26].

The exploratory PCA provided a complementary visualization of sample-level variation across the targeted gene panel. PC1 and PC2 together accounted for 60.3% of the total variance, and the plotted samples showed genotype- and treatment-associated variation. However, because the analysis included only three biological replicates per group and substantially more variables than samples, it was not used to test group separation or treatment-induced normalization. The PCA therefore illustrates variation within the targeted dataset but does not provide inferential evidence of global transcriptional remodeling or rescue.

The reduction in the NF-κB/inflammatory module score in APP/PS1 mice indicates lower expression of the genes included in this module. The Western blot data provided complementary protein-level evidence: APP/PS1-PBS mice showed higher total NF-κB p65 abundance than NonTg-PBS mice, whereas isoserine reduced total NF-κB p65 in APP/PS1 mice. This result was directionally concordant with the *Rela* transcript effect but did not directly demonstrate altered NF-κB pathway activation. This result is notable because NF-κB signaling occupies a complex position in AD biology. NF-κB can promote inflammatory activation and neuronal stress, but it can also participate in physiological plasticity and survival signaling depending on cell type, activation context, and disease stage [19,27,28]. However, because total p65 does not fully capture NF-κB activation state, future studies should assess phospho-p65, nuclear p65 localization, nuclear factor of kappa light polypeptide gene enhancer in B-cells inhibitor, alpha (IκBα) regulation, and downstream inflammatory targets.

The synaptic maintenance module also showed a significant APP/PS1 treatment effect and genotype × treatment interaction. This module included Dlg4, Ncam1, and Nptx2, genes linked to postsynaptic organization, cell adhesion, and excitatory synaptic function. PSD-95, encoded by Dlg4, is a major postsynaptic scaffold that organizes glutamatergic receptor complexes and supports synaptic signaling architecture [17,29]. Recent work further supports PSD-95 as a disease-relevant synaptic protein in AD, both as a marker of synaptic dysfunction and as a regulator of Aβ-related synaptic vulnerability [30,31]. NPTX2 is an activity-regulated neuronal pentraxin linked to excitatory synaptic function, parvalbumin interneuron circuit regulation, and cognitive vulnerability in AD [18]. More recent biomarker studies further implicate NPTX2 and related synaptic proteins in cognitive decline, prognosis, and cognitive resilience in AD-spectrum cohorts [32,33,34]. The Western blot data provided complementary evidence of altered postsynaptic protein abundance: APP/PS1-PBS mice showed lower PSD-95 levels than NonTg-PBS mice, whereas isoserine increased PSD-95 in APP/PS1 mice. Because *Dlg4* transcript abundance and PSD-95 protein abundance changed in opposite directions, the protein result does not directly validate the synaptic-maintenance module. Nevertheless, PSD-95 represents a plausible protein-level correlate of the treatment-associated behavioral effect.

Isoserine also modulated the glutamatergic signaling module in APP/PS1 mice. Although this module did not show a significant genotype × treatment interaction, the APP/PS1 treatment effect reached significance, indicating that isoserine affected glutamatergic signaling genes in diseased animals. Given the central role of glutamatergic signaling in hippocampal plasticity and the vulnerability of excitatory synapses to Aβ-mediated disruption, this module remains highly relevant to the behavioral phenotype [7,9]. The Western blot results provided complementary protein-level evidence. APP/PS1-PBS mice showed higher GluN2B abundance than NonTg-PBS mice, whereas isoserine reduced GluN2B in APP/PS1 mice. This directionally concordant *Grin2b*/GluN2B pattern supports an effect on this receptor subunit but does not establish normalization of NMDA receptor signaling. This interpretation is consistent with evidence that NMDA receptor signaling can contribute to both physiological plasticity and AD-associated excitotoxic stress depending on receptor localization, subunit composition, and disease context [35,36].

The immediate early gene module showed a strong trend toward treatment-induced modulation and genotype × treatment interaction but did not cross the *p* < 0.05 threshold. Immediate early genes such as Arc, Egr1, Egr2, Jun, and Srf play central roles in activity-dependent plasticity and memory consolidation [13,14,26]. Therefore, the trend observed in this module may still be biologically meaningful, particularly in the context of improved spatial memory retention. Nevertheless, we interpret this module cautiously and view it as a candidate pathway for future validation rather than a definitive mechanistic conclusion.

Taken together, the transcriptomic and protein-level data support a model in which isoserine improves cognitive performance by promoting synaptic resilience rather than by fully reversing the molecular state of the APP/PS1 brain. This distinction is important. APP/PS1 mice continue to express disease-driving transgenes and likely retain amyloid-associated pathology during treatment. Therefore, complete normalization of the transcriptional landscape would be biologically unlikely. The more plausible interpretation is that isoserine modifies selected disease-sensitive pathways sufficiently to improve memory retention. In particular, the combined decrease in NF-κB p65 and GluN2B, together with the restoration of PSD-95, suggests that isoserine attenuates inflammatory and glutamatergic dysregulation while strengthening postsynaptic integrity. This mechanism aligns with the broader concept that therapeutic benefit in AD may arise not only from reducing pathological burden but also from enhancing the ability of neuronal and synaptic networks to function in the presence of pathology [23,25,37,38].

This interpretation is also consistent with the limited but relevant literature on isoserine in the CNS. Previous work identified L-isoserine as a GAT3-selective substrate and showed that delayed treatment increased GAT3 expression and improved functional recovery after focal ischemic stroke in mice [20]. Although stroke and AD differ substantially in mechanism and temporal progression, both conditions involve synaptic dysfunction, altered inhibitory/excitatory balance, inflammatory responses, and circuit-level maladaptation. Our findings extend the biological relevance of isoserine by showing that it can improve memory and remodel synaptic and inflammatory pathways in an amyloid-associated AD mouse model. However, the present data do not establish whether the effects of isoserine in APP/PS1 mice depend on GAT3, altered GABA uptake, excitatory–inhibitory rebalancing, or additional mechanisms. To this end, we did not measure GAT3 expression or transport activity, extracellular GABA concentrations, or excitatory–inhibitory balance. Therefore, GAT3-dependent modulation remains a hypothesis rather than a demonstrated mechanism. L-isoserine also interacts with other extrasynaptic GABA transporters and the taurine transporter, so alternative transporter-dependent or peripheral mechanisms cannot be excluded. Future studies should quantify plasma and brain L-isoserine concentrations following oral administration, characterize its pharmacokinetic profile, assess GAT3 expression and GABA-transport activity, and determine whether genetic or pharmacological disruption of GAT3 prevents the behavioral and molecular effects of treatment. The existing literature itself notes that (S)-isoserine lacks optimal selectivity and brain permeability as an in vivo pharmacological tool. Future studies should directly test these possibilities.

This study has several limitations. First, the RT^2^ Profiler PCR Array interrogates a targeted panel of 84 synaptic-related genes rather than the entire transcriptome. This design strengthens hypothesis-driven interpretation but limits unbiased pathway discovery. Second, the sample size for the gene-expression analysis was modest, with three biological replicates per group. We therefore emphasized effect estimates, genotype × treatment interactions, descriptive effect-size patterns, and FDR-adjusted module-level analyses rather than relying solely on individual-gene significance. Third, we analyzed bulk tissue, which prevents us from assigning transcriptional changes to specific cell types. This limitation is particularly relevant for inflammatory genes such as Cebpb, Rela, and Nfkbib, which may reflect neuronal, astrocytic, microglial, or vascular contributions. Fourth, although the Western blot analyses provide complementary protein-level evidence, total NF-κB p65 does not fully capture NF-κB pathway activation. Future studies should assess phospho-p65, nuclear p65 localization, IκBα regulation, and downstream inflammatory targets. Future studies should assess soluble and insoluble Aβ species, plaque pathology, cell-type-specific transcriptional responses, synaptic protein localization, and electrophysiological measures of synaptic plasticity. In addition, although several individual genes reached nominal significance, no APP/PS1 isoserine treatment effect remained significant after FDR correction across the 84 target genes. Consequently, the individual-gene observations should be considered exploratory. The module-level analyses reduced the multiple-testing burden and identified FDR-significant coordinated effects, but these aggregate findings also require independent replication in larger cohorts and validation using complementary molecular approaches. Because we constructed the four literature-informed modules during exploratory analysis rather than preregistering them as confirmatory endpoints, the module-level findings should be regarded as hypothesis-generating and require validation in an independent cohort.

The molecular sample sizes were small and provided adequate sensitivity only for very large effects. A design-based sensitivity analysis indicated that a comparison with n=3 animals per group could detect standardized effects of approximately d≥3.07 with 80% power at α=0.05, whereas comparisons involving n=3 and n=4 animals could detect effects of approximately d ≥2.68. Consequently, the study had limited power to detect small or moderate molecular effects. We therefore interpret the individual-gene, heatmap, and PCA findings as exploratory. Although several module-level effects remained significant after FDR correction and the Western blot analyses identified large effects with confidence intervals excluding zero, these molecular findings require confirmation in larger independent cohorts.

Despite these limitations, the study provides converging evidence that isoserine improves spatial memory retention and is associated with disease-context-dependent molecular changes in APP/PS1 mice. The gene-level effect-size plots and exploratory heatmap and PCA illustrate treatment-associated expression patterns but do not establish transcriptional rescue, reproducibility, or statistically significant sample separation. The FDR-adjusted module analysis identifies coordinated changes in NF-κB/inflammatory, synaptic-maintenance, and glutamatergic-signaling gene-expression modules. Western blot analyses provide complementary protein-level evidence by showing reduced NF-κB p65 and GluN2B abundance and increased PSD-95 abundance in isoserine-treated APP/PS1 mice. Together, these findings support molecular remodeling without establishing pathway activation or a causal mechanism.

## 4. Materials and Methods

### 4.1. Animals and Experimental Groups

Non-transgenic (NonTg) and APP/PS1 transgenic mice were used in this study. Animals were originally obtained from The Jackson Laboratory (Bar Harbor, ME, USA; Stock No. 005864). All mice were maintained at 22 ± 2 °C under a 12 h light/12 h dark cycle, with free access to standard food and water.

All experimental procedures were conducted in accordance with Italian animal welfare regulations (D.M. 116/92), the European Directive (2010/63/EU), and the ARRIVE guidelines. All efforts were made to minimize animal pain and distress, including the use of non-invasive oral treatment and daily monitoring. No unexpected adverse effects related to L-isoserine administration were observed.

The animal study protocol was approved by the Institutional Animal Care and Use Committee (IACUC) of the Italian Ministry of Health (Ministero della Salute; protocol code: 938/2024-PR; approval date: 27 September 2024).

At 10 months of age, mice were allocated into four experimental groups based on genotype and treatment. The final number of animals that completed the behavioral testing and subsequent sacrifice at 12 months of age was as follows:

- NonTg-PBS group: n = 12 (9 males, 3 females)
- NonTg-ISO group: n = 14 (10 males, 4 females)
- APP/PS1-PBS group: n = 14 (10 males, 4 females)
- APP/PS1-ISO group: n = 14 (10 males, 4 females)

All animals included in the molecular analyses completed Morris water maze testing before tissue collection. For the RT^2^ Profiler PCR Array, three animals from each experimental group were selected randomly from the behavioral cohort, independently of Morris water maze performance. For the Western blot analyses, animals were selected through a separate random-selection procedure, also independently of behavioral performance. The PCR-array and Western blot subsets were therefore not designed as matched molecular samples (Figure 1).

### 4.2. Drug Treatment

Mice in the treatment groups received L-isoserine, corresponding to (S)-3-amino-2-hydroxypropionic acid (TCI, catalog no. I0828; CAS 632-13-3; purity > 98%), dissolved in drinking water at 1 g/L, equivalent to approximately 9.51 mM. Animals had continuous ad libitum access to the L-isoserine solution for eight weeks, beginning at 10 months of age and continuing until 12 months of age. Fresh treatment solution was prepared and supplied weekly. Control mice received drinking water without L-isoserine. We selected oral administration to provide a prolonged, non-invasive treatment regimen and to avoid the stress associated with repeated injections or the complications of chronic intracerebral delivery. We monitored body weight weekly throughout treatment to assess general tolerability.

Administration through drinking water was selected to provide continuous, non-invasive exposure over the eight-week treatment period and to avoid the repeated restraint and handling associated with daily oral gavage, which could affect behavioral outcomes. Individual water intake was not prospectively quantified. Assuming an approximate consumption of 8 mL per mouse per day, the formulation would provide approximately 8 mg of L-isoserine per mouse per day, corresponding to an estimated exposure of approximately 260–320 mg/kg/day over the observed body-weight range. This estimate should be interpreted cautiously because individual exposure may vary with body weight, sex, genotype, cage-level drinking behavior, day-to-day consumption, and bottle leakage or spillage. We did not perform pharmacokinetic measurements; therefore, neither the drinking-water concentration nor the estimated dose can be translated into a defined plasma or brain concentration.

### 4.3. Morris Water Maze and Sacrifice

Spatial learning and memory were evaluated using the MWM task over a total duration of 5 consecutive days. The test was administered during the final week of treatment (week 8) and mice were sacrificed immediately after the test, at 12 months of age. The experimental apparatus consisted of a circular pool (150 cm in diameter) filled with water maintained at a constant temperature of 25 °C. To prevent visual detection of the submerged platform, the water was made opaque by adding flour. The pool was conceptually divided into four quadrants, and distal visual cues (e.g., prominent posters) were positioned on the walls of the testing room to provide spatial reference points. An overhead video camera mounted above the pool was used to record and track all behavioral trials.

#### 4.3.1. Acquisition Phase (Days 1–4)

The acquisition phase was conducted during the first 4 days to assess spatial learning and orientation. A circular escape platform (14 cm in diameter) was submerged 1.5 cm below the water surface and fixed in a constant position within one of the four quadrants. Each mouse underwent 4 trials per day. For each trial, the mouse was released into the pool facing the wall from one of the three remaining quadrants, selected in a pseudo-random order. Animals were allowed to swim for a maximum of 60 s or until they successfully climbed onto the hidden platform. If a mouse located the platform, its escape latency was recorded, and the animal was allowed to remain on it for 10 s. If a mouse failed to find the platform within 60 s, a maximum latency of 60 s was recorded; the investigator then gently guided the animal to the platform without removing it from the water, allowing it to stay there for 10 s. Following each trial, the mouse was removed from the pool, gently towel-dried, and placed in a heated holding cage without bedding for a 25 s inter-trial interval before starting the next trial.

#### 4.3.2. Probe Trial (Day 5)

On the 5th day, a single probe trial was performed to evaluate long-term spatial memory retention. The submerged platform was completely removed from the pool. Each mouse was released into the water facing the wall from a pseudo-random starting location and allowed to swim freely for 60 s. The time spent in the target quadrant and latency to the first crossing of the former platform location were recorded and quantified via the video tracking system. Immediately following the completion of the behavioral testing on day 5, mice were sacrificed for subsequent tissue collection.

### 4.4. Total RNA Extraction

Cortical and hippocampal mouse tissues (~100 mg) were homogenized in 1 mL of chilled PureZOL reagent (Bio-Rad Laboratories, Inc., Hercules, CA, USA) utilizing an Ultra-Turrax device (IKA, Staufen, Germany). Total RNA was subsequently isolated in strict accordance with the manufacturer’s guidelines. To evaluate sample yields and purity, a NanoDrop spectrophotometer (SpectroStar Nano, BMG Labtech GmbH, Ortenberg, Germany) was employed; preparations exhibiting an A260/A280 absorbance ratio of 1.8–2.0 were classified as high-grade RNA [39].

### 4.5. cDNA Synthesis

Before reverse transcription, a genomic DNA elimination step was performed on 2.5 μg of total RNA. Subsequent complementary DNA (cDNA) synthesis was carried out utilizing the RT2 First Strand Kit (Qiagen GmbH, Hilden, Germany) in strict accordance with the provided protocol. Specifically, a 10 μL aliquot of the treated RNA suspension served as the template for each RT reaction [39].

### 4.6. Real-Time PCR for RT2 Profiler PCR Array

Quantitative amplification was carried out on a 7500 Real-Time PCR System (Applied Biosys-tems, Waltham, MA, USA) using the RT^2^ Profiler™ PCR Array Mouse Synaptic Plasticity (Cat. No. 330231, Qiagen GmbH, Hilden, Germany). Individual wells were loaded with 25 μL of reaction mix prepared with the RT2 SYBR^®^ Green qPCR Mastermix (Qiagen). The thermal profile consisted of a 10 min activation step at 95 °C, followed by 40 cycles of denaturation (95 °C for 15 s) and annealing/extension (60 °C for 1 min). Post-amplification melting curve assessment was performed to ensure specificity [39].

### 4.7. Data Analysis

We analyzed the RT^2^ Profiler PCR Array data using raw Ct values from three biological replicates per experimental group. We randomly selected the three animals in each group from the corresponding behavioral cohort without considering MWM performance. For each sample, we calculated the mean Ct of the five housekeeping genes, Actb, B2m, Gapdh, Gusb, and Hsp90ab1, and normalized each target-gene Ct value to this mean. We defined ΔCt as:

ΔCt = Ct_target_ − mean Ct_housekeeping_

We expressed normalized transcript abundance as −ΔCt, such that higher values indicate greater relative expression. Differences between group-level −ΔCt values correspond to log_2_ fold changes. We excluded the housekeeping genes and technical control wells, including the positive PCR control, reverse-transcription control, and mouse genomic DNA contamination control, from all target-gene analyses.

For each of the 84 target genes, we fitted a two-factor ordinary least-squares linear model that included genotype, treatment, and the genotype × treatment interaction as fixed effects. We used prespecified model contrasts to estimate four biologically relevant effects: the disease effect, calculated as APP/PS1-PBS minus NonTg-PBS; the isoserine treatment effect in NonTg mice, calculated as NonTg-ISO minus NonTg-PBS; the isoserine treatment effect in APP/PS1 mice, calculated as APP/PS1-ISO minus APP/PS1-PBS; and the genotype × treatment interaction, calculated as the difference between the isoserine treatment effect in APP/PS1 mice and the corresponding treatment effect in NonTg mice. For each gene and contrast, we calculated the effect estimate, 95% confidence interval, and two-sided *p*-value.

We controlled the false-discovery rate by applying the Benjamini–Hochberg procedure separately across the 84 target genes within each contrast family. We considered an adjusted q < 0.05 statistically significant. We report nominal findings with raw *p* < 0.05 but q ≥ 0.05 for transparency and interpret them as exploratory rather than as independently significant gene-level effects. Supplementary Table S1 provides the effect estimates, 95% confidence intervals, raw *p*-values, and FDR-adjusted q-values for all genes and contrasts.

To compare the transcriptional responses to isoserine across genotypes, we plotted the treatment effect in NonTg mice against the treatment effect in APP/PS1 mice for each gene. We used this treatment-effect scatter plot to visualize the direction and magnitude of genotype-dependent expression responses. Marker style did not encode nominal statistical significance.

To examine the directional relationship between disease- and treatment-associated expression effects, we plotted the disease effect against the isoserine treatment effect in APP/PS1 mice. Because both contrasts include the APP/PS1-PBS group with opposite signs, we treated this plot as a descriptive effect-size visualization and did not use the gene-wise correlation as an inferential test of transcriptional rescue or global normalization.

For the heatmap, we selected 14 genes based on effect magnitude, genotype-dependent expression patterns, representation of the functional modules, and biological relevance to synaptic and inflammatory processes. We used sample-level −ΔCt values and standardized each gene across the 12 biological replicates to obtain row-wise Z-scores. We used the heatmap as an exploratory visualization of expression patterns across individual animals and did not treat it as an independent statistical test.

Gene annotation in Figure 3 and Figure 4 and gene selection for the heatmap were performed post hoc for visualization purposes only. We did not apply a prespecified p-value, effect-size, or ranking threshold to determine which genes were annotated or included in the heatmap. Biological relevance referred to established roles in inflammatory regulation, activity-dependent transcription, synaptic organization, glutamatergic signaling, or intracellular signaling, based on the published literature and the functional categories represented in the targeted array. These visualization choices did not affect the gene-level statistical models, false-discovery-rate correction, or module-level analyses.

For principal component analysis, we used sample-level −ΔCt values from the 84 synaptic-related target genes after excluding housekeeping genes and technical controls. We retained 83 genes after removing genes with missing or zero-variance values that precluded their inclusion in PCA. Before performing PCA, we standardized each gene across samples to a mean of zero and a standard deviation of one so that genes with different expression ranges contributed comparably to the analysis. We used PCA exclusively as an exploratory visualization of the major sources of variation and did not perform an inferential test of group separation.

For the module-level analysis, we constructed four literature-informed exploratory gene-expression modules representing biological domains that were central to the study and directly represented on the targeted array. We did not preregister these modules as confirmatory endpoints before examining the dataset. After defining their composition, however, we held all gene assignments fixed before fitting the formal module-level statistical models and did not add or remove genes based on treatment-effect estimates, individual-gene *p*-values, or the resulting module-level significance.

The immediate-early-gene module included Arc, Egr1, Egr2, Jun, and Srf, based on their established roles in activity-dependent transcription and synaptic plasticity. The glutamatergic-signaling module included Gria4, Grin1, Grin2b, and Grm1, representing AMPA, NMDA, and metabotropic glutamate receptor signaling. The synaptic-maintenance module included Dlg4, Ncam1, and Nptx2, based on their roles in postsynaptic organization, cell adhesion, and excitatory synaptic maintenance. The NF-κB/inflammatory module included Cebpb, Rela, and Nfkbib, based on their direct involvement in NF-κB-associated transcriptional and inflammatory regulation.

We did not attempt to assign all 84 target genes to a module. We excluded genes with broad, pleiotropic, or ambiguous relationships to the four selected biological domains rather than forcing uncertain assignments. We assigned each included gene to only one module to avoid overlap and double counting.

For each biological sample, we calculated the module score as the arithmetic mean of the −ΔCt values of the genes assigned to that module. All constituent genes contributed equally to the module score. We did not apply weights based on baseline transcript abundance, variance, fold change, individual-gene statistical significance, or perceived biological importance. Because all −ΔCt values were expressed on the same logarithmic scale, differences between module scores represent the average expression difference across the genes assigned to each module.

We applied the same two-factor linear model and model contrasts used for the individual-gene analyses to each module score. We then applied the Benjamini–Hochberg procedure separately across the four modules within each contrast family and considered an adjusted q < 0.05 statistically significant. Supplementary Table S2 reports the module composition, effect estimates, 95% confidence intervals, raw *p*-values, and adjusted q-values.

Because the molecular analyses were exploratory and no reliable prior effect-size estimate was available for isoserine in APP/PS1 mice, we did not perform an a priori power calculation. A design-based sensitivity analysis showed that a two-sided comparison with n = 3 animals per group, α = 0.05, and 80% power could detect only standardized effects of approximately d ≥ 3.07. We therefore interpreted the individual-gene findings, selected-gene heatmap, PCA, and literature-informed module analyses cautiously and emphasized effect estimates, confidence intervals, multiple-testing correction, and the need for independent replication.

### 4.8. Protein Extraction and Western Blot Analysis

Half of the brain, with the cerebellum removed, was homogenized in 1 mL of T-PER™ Tissue Protein Extraction Reagent (Thermo Fisher Scientific, Waltham, MA, USA) supplemented with a protease and phosphatase inhibitor cocktail for 40 s using a mechanical homogenizer. The resulting homogenate was centrifuged at 12,000 rpm for 30 min at 4 °C to pellet tissue debris. Following centrifugation, the supernatant containing the total protein fraction was carefully collected and stored at −80 °C. Protein concentrations were determined using the Bio-Rad protein assay (Bio-Rad Laboratories, Hercules, CA, USA), with bovine serum albumin as the standard. A total of 30 µg of protein was loaded per lane for all Western blot experiments. Samples were then heated at 95 °C for 5 min, and equal amounts of protein were separated on 10–15% sodium dodecyl sulfate–polyacrylamide gel electrophoresis (SDS-PAGE) and subsequently transferred onto polyvinylidene difluoride (PVDF) membranes (Immobilon-P, catalog #88018; Thermo Fisher Scientific). Membranes were incubated overnight at 4 °C in a blocking solution (1× PBS containing 0.1% Tween-20 and 5% w/v non-fat dry milk) alongside primary antibodies sourced from Santa Cruz Biotechnology (Dallas, TX, USA). These included anti-NF-κB (sc-8008), anti-PSD-95 (sc-32291), and anti-GluN2B (sc-9057), all applied at a 1:500 dilution. Following the primary probe, blots were exposed to either goat anti-rabbit or bovine anti-mouse peroxidase-conjugated secondary IgG (1:2000; Jackson ImmunoResearch, Bar Harbor, ME, USA) for one hour at ambient temperature. To ensure uniform protein loading, the membranes were also probed with an antibody targeting glyceraldehyde-3-phosphate dehydrogenase (GAPDH) (sc-47724, 1:500; Santa Cruz). The blots were then developed as detailed in reference [40].

Western blot analyses included NonTg-PBS n=3, NonTg-ISO n=4, APP/PS1-PBS n=3, and APP/PS1-ISO n=4 biological replicates for each protein. We selected these animals independently and at random from the behavioral cohort without reference to MWM performance. The Western blot subset was not designed to match the animals used for the RT^2^ Profiler PCR Array.

For each protein, we assessed group differences using one-way ANOVA and quantified the omnibus effect using $R^{2}$. We evaluated variance homogeneity using the Brown–Forsythe test. We performed six pairwise comparisons among the four experimental groups and controlled the family-wise error rate using the Bonferroni procedure. For each comparison, we report the model-estimated mean difference, Bonferroni-adjusted simultaneous 95% confidence interval, and adjusted p-value. Supplementary Table S3 provides complete group-level descriptive statistics, omnibus results, and pairwise effect estimates.

### 4.9. Aβ42 Immunohistochemical Staining

Immunohistochemistry was performed on tissue sections (n = 3/group) using an Aβ42-specific antibody. Tissue sections were initially washed in Tris-Buffered Saline (TBS). To quench endogenous peroxidase activity, sections were incubated in a solution of 3% hydrogen peroxide in methanol, followed by washes in TBS. For antigen retrieval and unmasking of Aβ epitopes, sections were treated with 90% formic acid. Subsequently, sections were thoroughly washed in TBS to remove residual acid. To facilitate membrane permeabilization, sections were incubated in TBS-A (TBS containing Triton X-100). Non-specific binding sites were then blocked by incubating sections in TBS-B (TBS-A supplemented with BSA). Sections were incubated overnight at 4 °C with primary rabbit anti-beta amyloid 1-42 antibody (Merck, Cat# AB5078P, 1:1000) diluted in TBS-B. Following overnight incubation, unbound primary antibodies were removed by washes in TBS-A, followed by an incubation in TBS-B. Sections were subsequently incubated with a Biotinylated Pan-Specific Universal Antibody. After a wash in TBS-A, sections were incubated with Streptavidin/Peroxidase Complex, followed by a final wash in TBS-A. Signal detection was carried out by incubating sections in 3,3-diaminobenzidine (DAB) until optimal stain intensity was achieved, finally, the sections were rinsed with water and mounted on slides. Digital images of the stained sections were acquired and analyzed using NIS-Elements AR software (version 6.10.02). To quantify the amyloid pathology, the thresholding parameters were defined to selectively identify DAB-positive areas, and the percentage of Aβ burden was measured as the ratio of the Aβ-occupied area relative to the total region of interest (ROI) area.

## 5. Conclusions

Together, these findings identify isoserine as a candidate modulator of synaptic and inflammatory programs in AD and provide a rationale for further mechanistic studies aimed at defining how isoserine influences synaptic resilience, neuroinflammatory signaling, excitatory–inhibitory balance, and cognitive function.

## Figures and Tables

**Table 1:** Functional categories, constituent genes, and representative biological roles of genes discussed in the manuscript.

Functional Category	Genes	Representative Biological Roles
Immediate early genes	*Arc*, *Egr1*, *Egr2*, *Jun*, *Srf*	Activity-dependent transcription, neuronal plasticity, and memory-related gene regulation.
Glutamatergic signaling	*Gria4*, *Grin1*, *Grin2b*, *Grm1*	AMPA, NMDA, and metabotropic glutamate receptor signaling.
Synaptic maintenance	*Dlg4*, *Ncam1*, *Nptx2*	Postsynaptic organization, cell adhesion, and excitatory synaptic maintenance.
NF-κB/inflammatory signaling	*Cebpb*, *Rela*, *Nfkbib*	NF-κB-associated transcriptional control and inflammatory regulation.
Intracellular signaling	*Adcy1*, *Plcg1*, *Ppp1ca*, *Ppp2ca*, *Rheb*, *Kif17*, *Pim1*, *Ywhaq*	Second-messenger signaling, kinase/phosphatase regulation, receptor trafficking, and intracellular signal integration.
Endocannabinoid signaling	*Cnr1*	Cannabinoid receptor-mediated modulation of neurotransmission and synaptic activity.
Growth factor signaling	*Ngfr*, *Inhba*	Neurotrophin and growth-factor signaling associated with neuronal function and plasticity.
Housekeeping genes	*Actb*, *B2m*, *Gapdh*, *Gusb*, *Hsp90ab1*	Reference genes used to normalize RT^2^ Profiler PCR Array expression data.

Note: The immediate early gene, glutamatergic signaling, synaptic maintenance, and NF-κB/inflammatory signaling categories were used as literature-informed exploratory modules in the formal module-level analyses. The remaining categories are included to facilitate interpretation of genes discussed elsewhere in the manuscript. Mouse gene symbols are shown in italics. Housekeeping genes were used only for normalization.

## Data Availability

The original contributions presented in this study are included in the article/Supplementary Materials. Further inquiries can be directed to the corresponding authors.

## Supplementary Materials

The following supporting information can be downloaded at: https://www.mdpi.com/article/10.3390/cells15161487/s1, Figure S1: Chronic isoserine administration did not alter Aβ plaque burden; Table S1: Gene-level factorial analysis of the RT^2^ Profiler PCR Array; Table S2: Predefined module-level analysis; Table S3: Western blot sample sizes, descriptive statistics, and effect estimates.

## Abbreviations

The following abbreviations are used in this manuscript:

**Abbreviation**	**Definition**
AD	Alzheimer’s disease
Aβ	Amyloid-β
AMPA	α-Amino-3-hydroxy-5-methyl-4-isoxazolepropionic acid
ANOVA	Analysis of variance
APP/PS1	Amyloid precursor protein/presenilin 1 double-transgenic mouse model
Arc	Activity-regulated cytoskeleton-associated protein
cDNA	Complementary DNA
Cebpb	CCAAT/enhancer-binding protein beta
CI	Confidence interval
CNS	Central nervous system
CREB	cAMP response element-binding protein
Ct	Cycle threshold
Dlg4	Discs large MAGUK scaffold protein 4
DNA	Deoxyribonucleic acid
Erg1	Early growth response 1
Erg2	Early growth response 2
FDR	False discovery rate
GABA	γ-Aminobutyric acid
GAPDH	Glyceraldehyde-3-phosphate dehydrogenase
GAT3	GABA transporter 3
Gria4	Glutamate ionotropic receptor AMPA type subunit 4
Grin1	Glutamate ionotropic receptor NMDA type subunit 1
Grin2b	Glutamate ionotropic receptor NMDA type subunit 2B
GluN2B	NMDA receptor subunit GluN2B
Grm1	Glutamate metabotropic receptor 1
IACUC	Institutional Animal Care and Use Committee
IκBα	Inhibitor of nuclear factor κB alpha
ISO	Isoserine
Jun	Jun proto-oncogene
Kif17	Kinesin family member 17
MWM	Morris water maze
Ncam1	Neural cell adhesion molecule 1
Nfkbib	NF-kappa-B inhibitor beta
NF-κB	Nuclear factor kappa B
NMDA	*N*-Methyl-D-aspartate
NonTg	Non-transgenic
NPTX2	Neuronal pentraxin-2
PBS	Phosphate-buffered saline
PC1	First principal component
PC2	Second principal component
PCA	Principal component analysis
PCR	Polymerase chain reaction
Pim1	Pim-1 proto-oncogene, serine/threonine kinase
PSD-95	Postsynaptic density protein 95
PVDF	Polyvinylidene difluoride
qPCR	Quantitative polymerase chain reaction
Rela	RELA proto-oncogene, NF-kappa-B subunit
RNA	Ribonucleic acid
RT	Reverse transcription
SDS-PAGE	Sodium dodecyl sulfate–polyacrylamide gel electrophoresis
SEM	Standard error of the mean
Srf	Serum response factor
Ywhaq	Tyrosine 3-monooxygenase/tryptophan 5-monooxygenase activation protein theta
