# Supplementary material for: Isoserine Improves Spatial Memory and Remodels Synaptic and Inflammatory Gene-Expression Programs in APP/PS1 Mice
Source: Cells. 2026 Aug 18;15(16):1487. doi: 10.3390/cells15161487 (PMC13511242; doi:10.3390/cells15161487)
Supplement: Supplementary file 1 [file cells-15-01487-s001.zip › cells-4446637-supplementary/Supplementary Figure.pdf]

Supplementary Figures

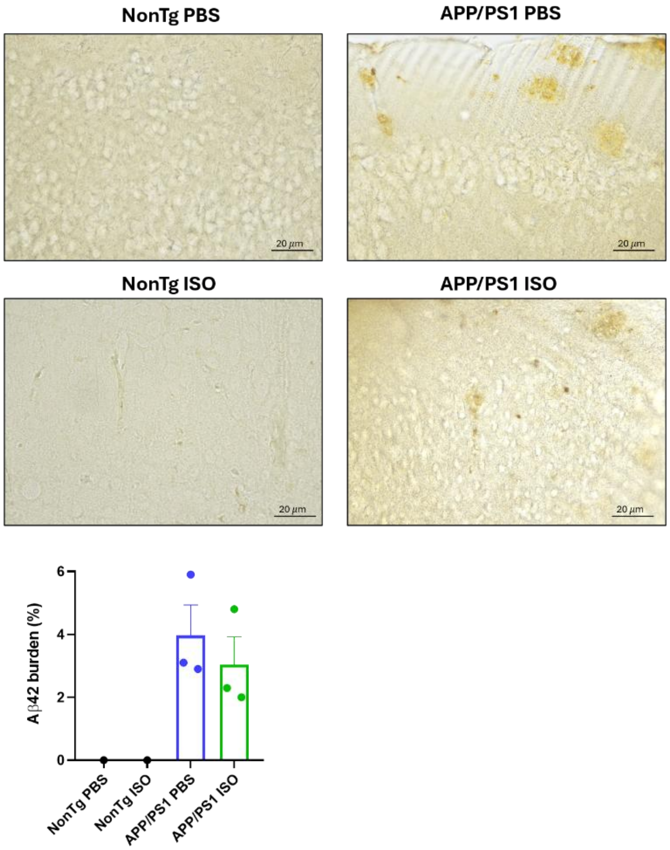

Supplementary Figure S1. Chronic isoserine administration did not alter Aβ plaque burden. Representative micrographs of Aβ-immunostained brain sections from mice of the indicated genotypes and treatment groups (n = 3/group). Quantitative analysis showed no significant difference in Aβ plaque burden between vehicle- and isoserine-treated APP/PS1 mice**.**
